# Radiosurgery for Five to Fifteen Brain Metastases: A Single Centre Experience and a Review of the Literature

**DOI:** 10.3389/fonc.2022.866542

**Published:** 2022-05-10

**Authors:** Susanne J. Rogers, Nicoletta Lomax, Sara Alonso, Tessa Lazeroms, Oliver Riesterer

**Affiliations:** Radiation Oncology Center KSA-KSB, Canton Hospital Aarau, Aarau, Switzerland

**Keywords:** radiosurgery, brain, metastasis, multiple, single isocenter, LINAC

## Abstract

**Purpose:**

Stereotactic radiosurgery (SRS) is now mainstream for patients with 1-4 brain metastases however the management of patients with 5 or more brain metastases remains controversial. Our aim was to evaluate the clinical outcomes of patients with 5 or more brain metastases and to compare with published series as a benchmarking exercise.

**Methods:**

Patients with 5 or more brain metastases treated with a single isocentre dynamic conformal arc technique on a radiosurgery linac were identified from the institutional database. Endpoints were local control, distant brain failure, leptomeningeal disease and overall survival. Dosimetric data were extracted from the radiosurgery plans. Series reporting outcomes following SRS for multiple brain metastases were identified by a literature search.

**Results:**

36 patients, of whom 35 could be evaluated, received SRS for 5 or more brain metastases between February 2015 and October 2021. 25 patients had 5-9 brain metastases (group 1) and 10 patients had 10-15 brain metastases (group 2). The mean number of brain metastases in group 1 was 6.3 (5-9) and 12.3 (10-15) in group 2. The median cumulative irradiated volume was 4.6 cm^3^ (1.25-11.01) in group 1 and 7.2 cm^3^ (2.6-11.1) in group 2. Median follow-up was 12 months. At last follow-up, local control rates per BM were 100% and 99.8% as compared with a median of 87% at 1 year in published series. Distant brain failure was 36% and 50% at a median interval of 5.2 months and 7.4 months after SRS in groups 1 and 2 respectively and brain metastasis velocity at 1 year was similar in both groups (9.7 and 11). 8/25 patients received further SRS and 7/35 patients received whole brain radiotherapy. Median overall survival was 10 months in group 1 and 15.7 months in group 2, which compares well with the 7.5 months derived from the literature. There was one neurological death in group 2, leptomeningeal disease was rare (2/35) and there were no cases of radionecrosis.

**Conclusion:**

With careful patient selection, overall survival following SRS for multiple brain metastases is determined by the course of the extracranial disease. SRS is an efficacious and safe modality that can achieve intracranial disease control and should be offered to patients with 5 or more brain metastases and a constellation of good prognostic factors.

## Introduction

The treatment landscape for patients with brain metastases has transformed in the past 15 years. A nihilistic approach used to prevail due to the associated mean survival of 3-4 months (1). Patients mostly presented with a poor performance status due to large, symptomatic brain metastases and were treated with whole brain radiotherapy (WBRT). WBRT can achieve symptomatic relief but without significant tumour control and cause of death in such patients is frequently neurological (2). A positive correlation between radiotherapy dose, local control rate and overall survival in patients with brain metastases has been established (3) and stereotactic radiosurgery (SRS), which is high dose irradiation to small target volumes, can achieve long lasting local control of brain metastases with minimal toxicity in patients eligible for this approach. The mean and maximum biologically equivalent doses in brain metastases with SRS are 3 and 5 times greater respectively than with 10 x 3Gy WBRT (4) and by achieving intracranial disease control and avoiding a neurological cause of death, can even increase survival as compared with WBRT (5, 6).

With earlier detection of small brain metastases through increased access to magnetic resonance (MR) imaging, the development of disease-specific prognostic indices, identification of druggable molecular targets and widespread adoption of immunotherapy, the prognosis for subgroups of patients with brain metastases and controlled extracranial disease has increased dramatically. Overall survival of up to four years in patients with more than four brain metastases from EGFR- and ALK-mutated non-small cell lung cancer following radiosurgery has been reported (7). Consequently, the management of brain metastases in patients with a better prognosis should be individualized and intensified in patients with a constellation of positive prognostic factors.

Radiosurgery has developed from a time-consuming, labor-intensive therapy only viable for patients with very few brain metastases to a practically manageable option for patients with multiple brain metastases (MBM). The definition of multiplicity is currently unresolved and, according to the National Comprehensive Cancer Network (NCCN), extends to ‘all patients who would profit from radiosurgery as compared with whole brain radiotherapy’ (8). Historically, patients were highly selected for brain radiosurgery due to the limited access to radiosurgery platforms. Three or four brain metastases are, or at least were, generally the upper limit for radiosurgery in many centers (9). This is partly due to the lengthy duration of sequential treatment of MBM, the time-intensive quality assurance and constraints by healthcare systems. Furthermore, the radiosurgery community was slow to adopt radiosurgery for MBM due to safety concerns. The potential toxicity from the cumulative irradiated volume when treating MBM was uncertain, and it was argued that the integral dose to the brain was likely to be as high as with WBRT but this has been disproven (10). Publication of the large, multicentre cohort JLGK0901 study, which reported that overall survival and most secondary endpoints following radiosurgery for 5-10 brain metastases were not inferior to 2-4 brain metastases (11), has been practice changing. The same group also reported that clinical outcomes for patients with 10-15 brain metastases were equivalent to patients with 2-9 brain metastases when treated with radiosurgery (12). The number of patients with MBM referred to our centre for radiosurgery has steadily increased in recent years. The optimal therapy for five or more brain metastases is still controversial (13) and represents the focus of this study. The purpose of this work was to evaluate the clinical outcomes of our cohort of patients, to discuss the technique and to provide a systematic overview of the current literature.

## Methods

### Inclusion Criteria

The prospectively-maintained institutional database was searched for consecutive patients who received radiosurgery to five or more intact brain metastases in a single treatment course between 1^st^ December 2015 and 1^st^ November 2021. Ethics approval was obtained (EKNZ 2019-01705) and patients who, at the time of treatment, declined general consent to participate in future research were not included. All patients were presented at a multidisciplinary neuro-oncology tumour board where a recommendation for SRS was made.

### Treatment Planning Technique

A CT with contiguous 0.6mm slices (14) was performed in a customized radiosurgery mask (Brainlab, Germany) and a 1.5 T T1-Gad MPR planning MRI scan were obtained on the same day. The CT and MRI were fused rigidly and then again with deformable registration to correct any distortion in the MRI. Following autosegmentation of the organs at risk (Brainlab Elements), the contrast-enhancing brain metastases were contoured and a 1mm planning target volume (PTV) margin was added to each, unless they were located in the brainstem when no PTV margin was added. For metastases more than 4cm off-axis and of volume <0.07 cm^3^ (equivalent to a diameter of approximately 5 mm), a 1.5 mm or sometimes 2mm margin was applied to correct for any rotational inaccuracy.

The prescription dose was 20 Gray (Gy) in a single fraction to 98–99% of the PTV (15), with a maximum dose between 125 and 143% (equivalent to prescribing to the 70–80% isodose surface (%IDS) when normalized to the maximum point dose). The structure ‘brain minus GTV’ was created and if more than 10 cm^3^ of this ‘organ at risk’ (OAR) received 10 Gy per metastasis, the dose was reduced to 30 Gy in 5 fractions, allowing 20 cm^3^=V20Gy, using the same prescription isodose. Metastases greater than 2cm in diameter or located in eloquent cortex were also treated with hypofractionated stereotactic radiotherapy (hfSRT). In the brainstem, small metastases were treated with a single fraction of 18 Gy. Treatment plans were generated using Elements Multimets v1.5 and v2.0 (Brainlab, Germany).

### Treatment Delivery

Treatment was delivered with non-coplanar dynamic conformal arcs (DCA) on a Truebeam STx linear accelerator (linac) with Novalis Radiosurgery platform (Brainlab/Varian) with high definition MLC leaves (2.5 mm) and a 6 degrees of freedom (DoF) couch. 4mg daily prophylactic dexamethasone for 3 days was prescribed for metastases with a cumulative volume in excess of 1 cm^3^.

An accurate patient set-up and treatment delivery was ensured using the Brainlab stereoscopic Exactrac kV x-ray 6D image-guided radiotherapy system. Before delivering each DCA, the stereoscopic radiographic images were matched to the reference digital radiographs reconstructed from the planning CT data set. Before delivery of the first arc, translational and rotational corrections were applied using the 6DoF couch. Verification images were taken before each further arc and corrections applied for translational shifts greater than 0.5mm and rotational shifts greater than 0.5 degrees.

Follow-up MRIs were performed every 3-months after radiosurgery and time to local recurrence, nodular leptomeningeal recurrence, new brain metastases and radionecrosis were calculated from the date of last radiosurgery. Patient follow-up was censored at death or last contact up to 31^st^ October 2021.

### Statistical Analysis

Kaplan–Meier analysis was utilized to calculate the actuarial local control rate and overall survival rates, otherwise descriptive statistics were applied. Patients were censored at death for the local control analysis.

### Literature Review

Terms for the literature search in Pubmed with no time limit and up to 31^st^ October 2021 were “radiosurgery”, “metastasis”, “brain” and “multiple”. Original reports published in English, French or German of patients who received radiosurgery for 2 or more brain metastases were included if sufficient data regarding outcomes of patients with 5 or more brain metastases were available. Dosimetric evaluations without clinical data were excluded, as were reviews of the technical or clinical issues. Reports pertaining mainly to quality of life, health economics, toxicity and non-SRS therapies were also excluded. No filters, limits or automation were used. No assumptions were made as to missing data, which are presented as ‘not reported’ (NR). The review was performed following the PRISMA 2020 guidelines however a formal meta-analysis was beyond the scope of this work.

## Results

Radiosurgery for 5 or more brain metastases was delivered to 37 patients between February 2017 and October 2021. 5-9 brain metastases were treated in 26/37 (70%) (group 1) and 10 or more brain metastases were irradiated in 11/37 patients (30%) (group 2). One patient in group 1 moved abroad for further treatment and was lost to follow-up and one patient in group 2 was not included in the final analysis as only one of the five hypofractionated stereotactic radiotherapy fractions could be delivered, thus 25 and 10 patients were evaluated in groups 1 and 2 respectively. All patients were in recursive partitioning analysis (RPA) group 2 as none had a single metastasis and all patients had a minimum Karnofsky Performance Status of 70%. Median patient follow-up in group 1 was 12.1 months and in group 2 was 15.6 months.

Patient demographics are shown in **Table 1**. Patients in groups 1 (5-9 BM) and 2 (10-15 BM) were similar in terms of age and performance status. Two thirds of each group had non-small cell lung cancer but driver mutations were rare (**Table 1**). On average, patients in group 2 had twice as many BMs as those in group 1 (mean 12.3 vs 6.3). More patients in group 2 had a synchronous diagnosis of BM (within 4 weeks of the primary tumour, often as part of tumor staging), and thus a shorter mean interval to diagnosis of the BMs (median 0.7, mean 10.9 months) than patients in group 1 (median 3.9, mean 18 months). No patients in group 2 had had prior WBRT, whereas 2/25 (8%) patients in group 1 had previously received therapeutic WBRT. More than two thirds of patients received concomitant systemic therapy as summarized in **Table 1**. With regard to dose prescription, 5 of 25 patients (20%) in group 1 received a combination of single fraction and hfSRT in the same treatment course and in group 2, 30% (3 of 10 patients) required this combined prescription approach.

**Table 1:** Patient demographics.

Patient Characteristics	Group 1 (5-9 BM)	Group 2 (10-15 BM)
Number of patients	25	10
Gender M:F	14: 11	3: 7
Age (yrs) mean (range)	65.4 (50-80)	62.5 (51-69)
Mean Karnofsky Performance Status (%), mean (range)	86.5 (70-100)	88.3 (80-90)
Adenocarcinoma of the lung: other	17: 8	7: 3
-Targetable TK mutation Y:N	2: 23 (8%)	2:8 (20%)
Mean number of BMs per patient (range)	6.3 (5-9)	12.3 (10-15)
No. of patients with a ds-GPA score for the primary	19/25	10/10
Median ds-GPA (range)	1.5 (0-2.5)	1.5 (1-2.5)
Prior irradiation of other BM Y: N	5: 20 (20%)	1: 9 (10%)
-SRT/SRS	3/5	1/1
-WBRT	2/5	
Synchronous: metachronous BM	16: 9 (64%)	8:2 (80%)
Time to BM from diagnosis of primary in months median (range)	3.9 (0-187.5)	0.7 (0-95.5)
Extracranial metastases Y:N	20:5 (80%)	10:0 (100%)
Synchronous systemic treatment Y:N	10:15 (66.7%)	8:2 (80%)
-Chemotherapy	3/10	2/8
-Immunotherapy	2/10	0/8
-Immunochemotherapy	2/10	4/8
-Tyrosine kinase inhibitor	3/10	2/8
Symptomatic BM Y: N	1:24 (4%)	2:8 (20%)
No. of patients with combined SRS/hfSRT: single fraction SRS only prescribed in same treatment course	5:20 (20%)	3:7 (30%)

BM, brain metastasis; SRS, stereotactic radiosurgery; hfSRT, hypofractionated stereotactic radiotherapy.

**Table 2** represents the dosimetric features of the radiosurgery plans for multiple brain metastases. The brain metastases were small with a cumulative total volume of 4.6 cm^3^ in group 1 and 7.2 cm^3^ in group 2 respectively. The plan quality as measured by the conformity and gradient indices were comparable in the two groups.

**Table 2:** Dosimetric features of SRS plans for multiple metastases.

Plan Characteristics	Group 1 (5-9 BM)	Group 2 (10-15 BM)
Median GTV per metastasis, cm^3^ (range)	0.2 (0.06-1.47)	0.32 (0.04-0.56)
Median PTV per metastasis, cm^3^ (range)	0.9 (0.20-3.08)	0.6 (0.22-0.98)
Cumulative total PTV per patient, cm^3^ Median (range)	4.6 (1.25-11.01)	7.2 (2.6-11.1)
Mean number of isocentres per patient (range)	2.3 (1-4)	3.0 (2-4)
Mean distance of metastasis from isocentre, cm (range)	2.9 (1.72-3.88)	3.2 (3.08-3.88)
Mean inverse Paddick Index per BM (range)	1.3 (1.15-1.54)	1.5 (1.41-1.74)
Mean Gradient Index per BM (range)	3.8 (2.54-4.88)	4.0 (3.47-5.3)
Mean number of arcs per isocentre (range)	7.8 (3-10)	8.8 (4-10)
Mean number of monitor units per Gray (range)	279.6 (212-539)	318.9 (169-577)

BM, brain metastasis.

**Figure 1:**
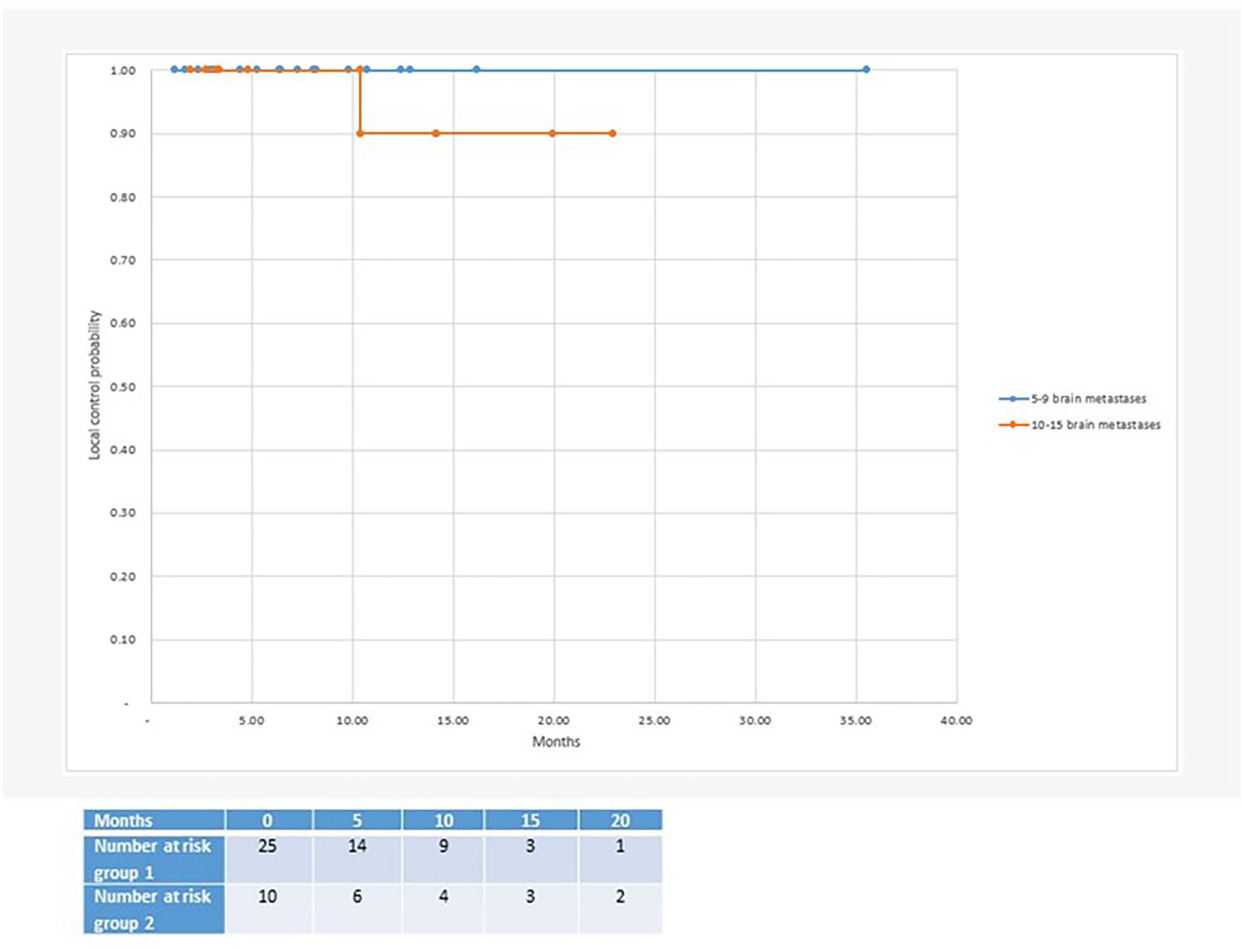
Median local control after radiosurgery for 5 or more brain metastases: Group 1 with 5-9 brain metastases (100% local control at 35 months follow-up) and group 2 with 10-15 brain metastases (90% local control at 23 months follow-up).

**Figure 2:**
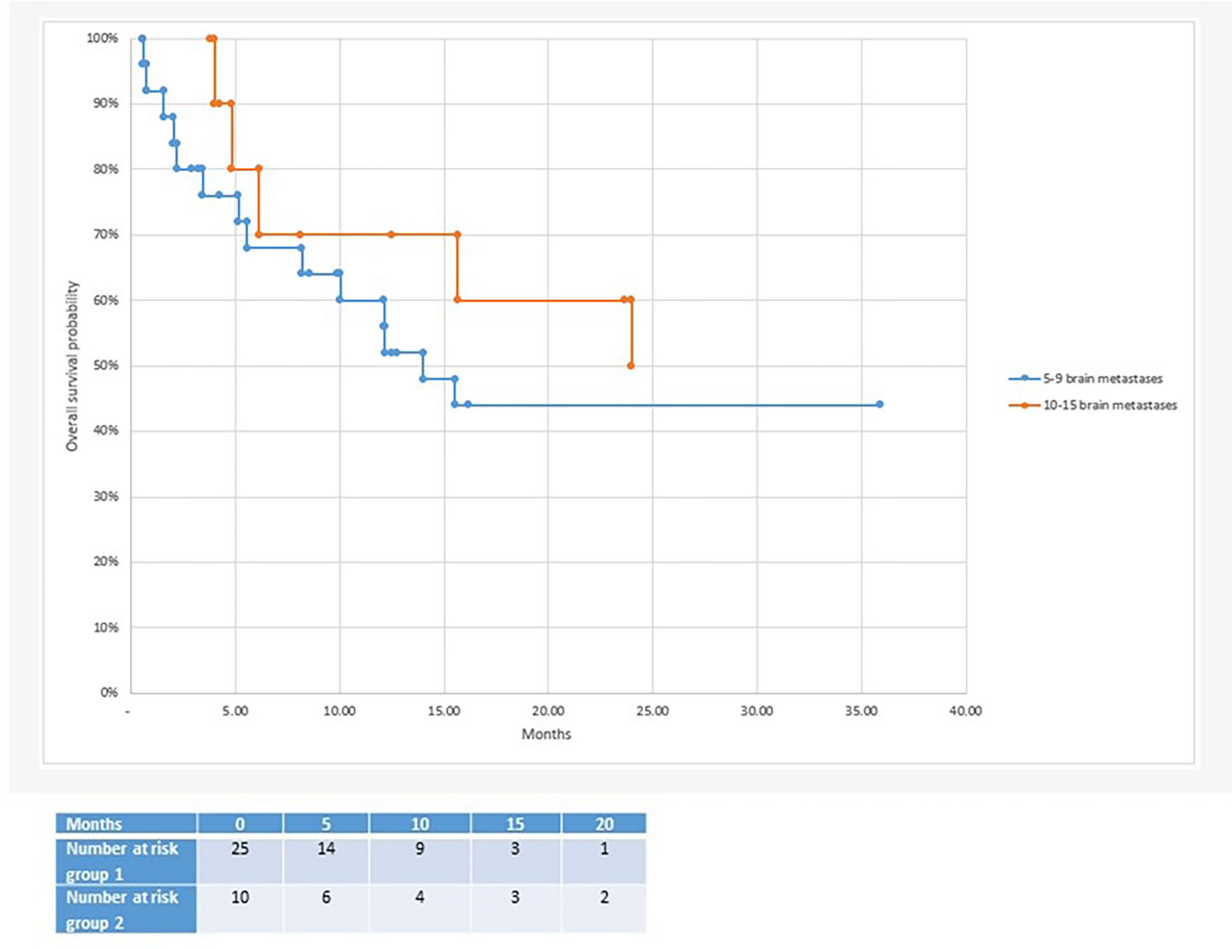
Median overall survival after radiosurgery: 10 months in the 5-9 brain metastases group vs 15.7 months in the 10-15 brain metastases group.

**Figure 3:**
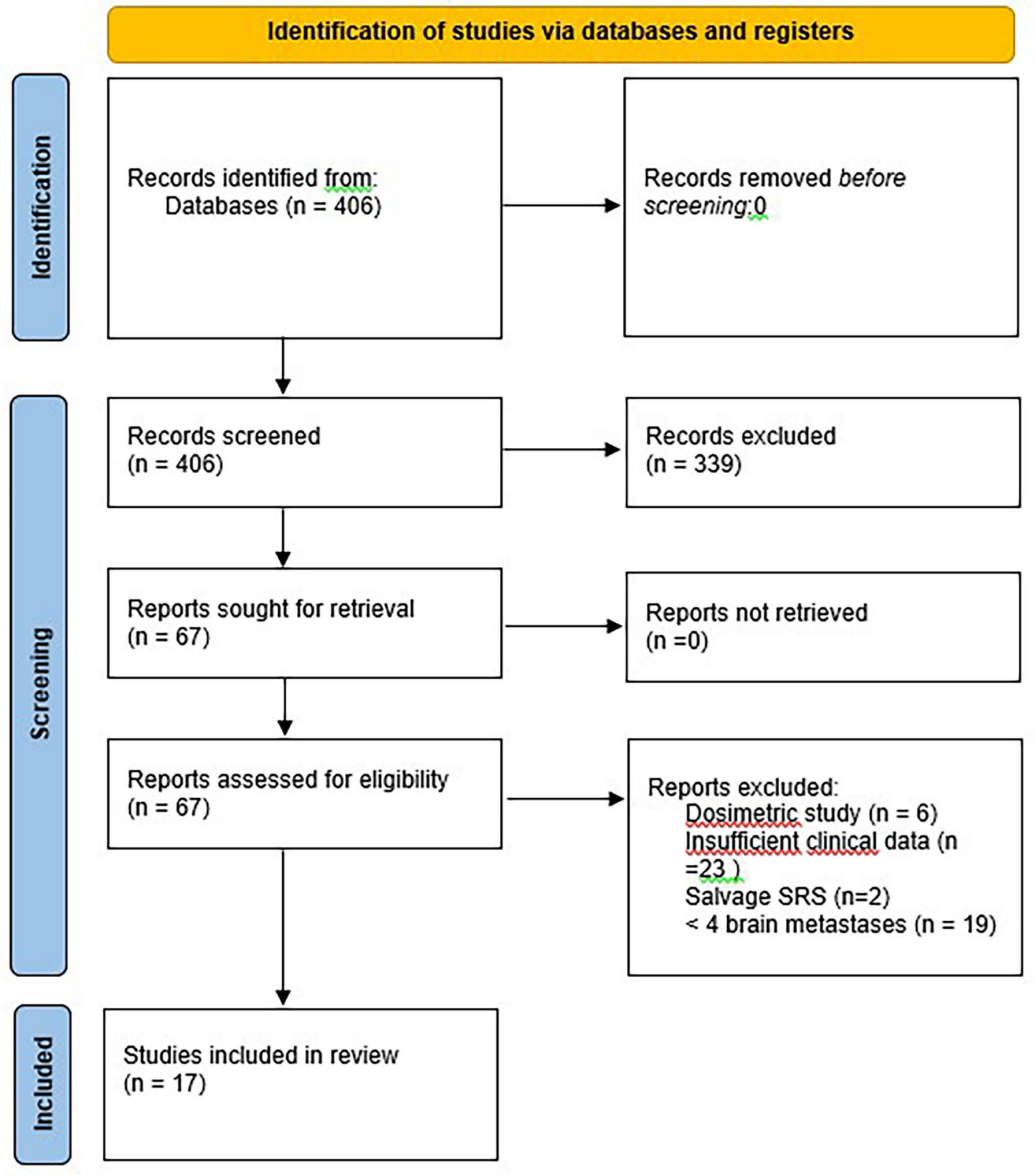
Flow diagram representing the number of records identified and reasons for exclusion.

With regard to clinical outcomes, local control was evaluated per lesion and per patient. At a median follow-up of 12.1 and 15.6 months, the local control rates at last follow-up approximated 100% in both groups (**Figure 1** per patient and **Table 3** per lesion). Metabolic activity was detected in two initially larger metastases on FET-PET CT scan 1 year after hfSRT as discussed below. There were no reported toxicities according to CTCAE v5.0. Median overall survival was 10 and 15.9 months in groups 1 and 2 respectively (**Figure 2**), which exceeds that reported in the literature to date. 17 publications were included in the literature review (**Figure 3**). 10/17 described outcomes following SRS for patients with five or more brain metastases and seven publications with patients with four or more brain metastases were included, as the cut-off for the definition of MBM is arbitrary (**Table 4**). Only two series used a linac to deliver SRS (25, 30). Considering all patients and the data provided, the median number of brain metastases irradiated per patient was 7 and the median cumulative tumour volume per patient was 5.7cm^3^. The median local control rate at 1 year was 87% and the median overall survival was 7.6 months.

**Table 3:** Clinical outcomes following SRS.

Clinical outcome	Group 1 (5-9 BM)	Group 2 (10-15 BM)
Median follow-up (range) in months	12.1 (0.6-37.5)	15.6 (3.8-24)
Local failure at last follow-up (per BM)	0/159 (0%)	2/123 (0.02%)
Distant brain failure (new BM) Y:N	9:16 (36%)	5:5 (50%)
Time to distant brain failure in months median (range)	5.2 (2-24)	7.4 (2-22.5)
Brain metastasis velocity (no. of new BM/year)		
-at first distant brain failure	9.7	11
-at time of last follow-up	1.9	2.7
Incidence of leptomeningeal relapse	1:24 (4%)	1:9 (10%)
Brain irradiation at DBF Y:N	9:16 (36%)	5:5 (50%)
-hfSRT/SRS	7/9 (1*/7) (78%)	1*/5 (20%)
-WBRT	2/9 (22%)	5/5 (100%)
Extracranial disease progression Y:N	15:10 (60%)	7:3 (70%)
Therapy at extracranial disease progression (several possible)	15/15 (100%)	3/7 (30%)
-SBRT	2/15	1/3
-Surgery	1/15	0/3
-Chemotherapy	0/15	1/3
-Tyrosine kinase inhibitor	7/15	1/3
-Immunotherapy	5/15	0/3
-Best supportive care		1/3
Median overall survival (range) in months	10.0 (0.6-35.9)	15.7 (3.8-24)
Deceased at last follow-up	14/25 (56%)	5/10 (50%)
Neurological cause of death (no. of patients)	0/25	1/10

NR, not reached; BM, brain metastasis; DBF, distant brain failure; hfSRT, hypofractionated stereotactic radiotherapy; *SRS at second DBF; WBRT at third DBF.

**Table 4:** Summary of the literature pertaining to clinical outcomes after radiosurgery for four or more brain metastases.

	No. of		Platform	Median no. of BM (range)	Median follow-up (mths)	Median total treatment volume per patient (cm^3^) (range)	PTV per metastasis (cm^3^) Mean, (range)	Prescribed dose (Gy) Mean(range)	1 yr LC (%)	DBF 1yr (%) Med. time to DBF (mths)	Median OS(mths)	Criteria/Comments
	BM	pts										
Nam et al., 2005 (16)	≥4 BM	46	Gamma knife	Mean 4.24 (127)	13.3	Mean 8.38 (0.87-104)	1.92	17.9 (12-30)	69.5	20.9	5.4	
Bhatnagar et al., 2006 (17)	≥4	205	Gamma knife	5 (4-18)	Mean 8	6.8 (0.6-51)	NR	16	71	43 9	8	46% SRS in combination with WBRT 38% SRS as salvage after WBRT
Kim et al., 2008 (18)	≥10	26	Gamma knife	16.6 (10-37)	NA	10.9 (1.0-42.2)	NR	15(9-23)	79.5% @ 6 mths	26.9 6 mths	7.8	
Chang et al., 2010 (19)	6-10 11-15 >15	58 17 33	Gamma knife	NR	10.7 12.3 8	NR	NR	NR	83 92 89	11 8 6 (p=0.028)	10 13 8	
Lee et al., 2011 (20)	4-14	36	Gamma knife	7 (4-14)	4.5	1.2 (0.002-12.6)	NR	17.8 (12-22)	84.2 @ 9 mths	22.2 4	9.1	Median KPS 90 80.6% no prior WBRT 70% dose if WBRT < 2 yrs
Grandhi et al., 2012 (21)	≥10	61	Gamma knife		4	4.86 (0.14 -40.21)	0.64 (0.01–2.87)	16	48.	77.6 3	4.5	77% KPS 90-100 37.7% no prior WBRT
Mohammadi et al., 2012 (22)	≥5	170	Gamma knife	6 (5-20)	6.2	3.2 (0.2-37.2)	Max. diameter 1.8 (0.5-5.1)	NR	97	40 (crude) 2.1	6.7	SRS as salvage in 110/170 (65%) patients
Rava et al., 2013 (23)	≥10	53	Gamma knife	11 (10-34)	NR	NR	NR	16.6	86.8	90 3	<10BM: 6.8 >10BM: 5.8	KPS >70, 36% no prior WBRT PTV = GTV + 1-2mm
Salvetti et al., 2013 (24)	5-15 5-9	96 10-15	Gamma knife	7 (5-15)	4.1	6.12 (0.42-57.83)	0.26 (0.007-46.54)	20 (14-36.4)	84.8	41	4.8 3.4(NS)	All histologies except SCLC and CUP, KPS>70, 53% no prior WBRT
Yamamoto et al., 2014 (11)	5-10	208	Gamma knife	6	12	3.54 (NR-13.90)	Max. diameter 1.62 (0.08-2.97)	<4cm^3^22 >4cm^3^20	93.5	64	10.8	Max 3cm diam/10cm^3,^ cumulative tumor vol. <15cm^3^, KPS>70, no prior WBRT
Frakes et al., 2015 (25)	≥5	28	Linac + Exactrac		6.3	3.7 (0.6-16.9)	0.34 (0.01-12.5)	24 (15-24)		57.1% @med 3 mths (1-15)	7.6 from SRS	Exclusively melanoma patients
Greto et al., 2016 (26)	>4 BM	11	Gamma knife	NR	7.2	NR	Mean PTV 0.39 (0.006-1.86)	20.3 (11-24)	95	3	72.4% @1yr	
Knoll et al., 2016 (27)	>4 BM	70	Gamma knife or Cyberknife	NR	NR	1.8 cm^3^	NR	NR	96.8 @ 6 mths	NR	8.5 (4.4-12.9)	
Yamamoto et al., 2019 (12)	2-9 >10	467 467	Gamma knife	NR	6.1 (1.2-11.8)	Mean 10.4 (0.06-115.3) 9.8 (0.15-81.4)	Mean of largest tumour 5.8 (0.04-57.8) 5.3 (0.03-65)	20.9 (10-25) 21.1 (12-25)	Timepoint? 92 96.2		7.1 6.9	
Hamel-Perreault et al., 2019 (28)	5-9 >10	81 22	Gamma knife	7 (5-19)	13 (1-35)	2.0 (0.06-28.0)	1.1 (0.02-16)	20 (16-25)	79% at 6 months	53% at 6 months	6 (1-58)	
Susko et al., 2020 (29)	≥- 10	143	Gamma knife	13 (11-17)	7.4 (2.7-15.9)	4.1 (2-9.9)	NR	19 (18-19)	96.8% (primary) 83.6% (salvage)	80.2 (primary 80.8 (salvage)	11.7 (primary) 7.4 (salvage)	
Alongi 2021 (30)	2-22	172	2.5mm MLC Linac SI-VMAT	4 (2-22)	20	5.7 (0.3–74.3)	0.2 (0.08–24.4)	mean 9 (4–25) 1 x15 to-5 x 6	86.7	80.6	12 (3-33)	Single isocentre
KSA series 2022	5-9 10-15	25 10	2.5mm MLC Linac + Exactrac SI-DCA	NR	12.1 15.6	4.6 (1.25-11.01) 7.2 (2.6-11.1)	0.5(14.04) 0.2(3.98)	20 (18-29) 1 fr 1 x 18 to 5 x 6	100 90	36% 50% 5.2 7.4	10 (0.6-35.9) 15.7 (3.8-24)	All histologies except SCLC (50% adeno NSCLC) KPS >80, 6% prior WBRT

Pts, patients; fr, fraction; NR, not reported.

## Discussion

Numerous comparative planning studies of radiosurgery for MBM have been published, however there are fewer reports of clinical outcomes of patients treated with 5 or more brain metastases, and very few with a linac (25, 31). This observation prompted us to evaluate our cohort of patients and to benchmark these real-world data from routine clinical practice against the literature.

The local control rates per patient and per lesion in excess of 90% at 12 months in both groups confirm accurate irradiation of the small target volumes and reflect the greater efficacy of SRS for small metastases (32). One patient with 13 BMs had local progression of 1 brain metastasis after initial hfSRT. 11 brain metastases were small and could be treated with a single fraction, but two were located in the eloquent motor cortex. As the patient was symptomatic with focal seizures affecting his dominant arm and the PTV volume was 3.98 cm^3^, these two metastases were treated in a separate volume with hfSRT to 30 Gy in 5 fractions. After 10.4 months, an MRI scan was reported to show enlargement following an initial good partial response and thus possible tumour progression of the largest metastasis. FET-PET imaging confirmed metabolic activity of vital tumour cells, rather than radionecrosis, in the two metastases treated with hfSRT as well as a third metastasis. In the context of extracranial progressive disease and to minimise the risk of radionecrosis, salvage WBRT with hippocampal avoidance was performed. Neurological death was reported in one patient in group 2 who succumbed during a generalized epileptic seizure 16 months after SRS for MBM whilst hospitalized and receiving best supportive care for extracranial disease progression. In the other 34 patients, extracranial disease progression was the cause of death.

As early as 2000, Suzuki et al. reported good safety and local control data but a mean survival of only 11 weeks for 24 patients treated with SRS for more than 10 brain metastases. Early reports emphasised the lack of difference in OS as compared with patients treated with SRS for more or fewer than 4 brain metastases (26). At a median follow-up of 12 months, 50% of all 35 patients with 5 or more brain metastases in this series are alive (approximately 50% of each group). The prolonged median overall survival of up to 16 months in our series demonstrates appropriate identification of patients with MBM likely to benefit from radiosurgery. Patient selection is often levelled as a criticism of single centre series, however is necessary in the setting of SRS for MBM to personalize therapy and to optimize use of resources. The management of such patients requires particular consideration of their prognosis due to extracranial as well as the intracranial tumour situation, with for example differentiation of visceral from non-visceral metastases (17). The disease-specific graded prognostic assessment (ds-GPA) scores in groups 1 and 2 were low (median 1.5) as more than four brain metastases receives a score of zero, furthermore most patients had extracranial disease and driver mutations were infrequent in these small patient groups. Sperduto et al. determined a median survival of 12 months with a ds-GPA score of 1.5-2 in patients with adenocarcinoma of the lung, which is in the order of the median 10-16 months in this study. Several patients are alive with an overall survival of 24-35.9 months, well in excess of that predicted from their ds-GPA scores. Nagtegaal et al. found a correlation between actual and predicted overall survival according to ds-GPA score in a cohort of over 350 patients with 0-10 brain metastases (33), except for a worse than predicted OS in the subgroup of patients with adenocarcinoma of the lung and a ds-GPA score of 2.5-4.0. Recently, the ds-GPA could not be validated in a cohort of patients with melanoma, putatively due to the effects of immunotherapy and targeted therapies (34). This group suggested a novel approach to predictive scoring using a combination of tumour volume, timing of onset and any systemic therapies, which reflects the continual personalization of therapy for patients with brain metastases (34).

It has been suggested that the number of brain metastases is a surrogate for the disease burden, rather than being prognostic per se (27) however brain metastases velocity (BMV), that is to say the number of new brain metastases developing per year, is predictive of outcome (35). A statistically longer interval to new brain metastases for patients with >2 BMs relative to patients with >15 BMs at SRS has been shown (35). Time to distant brain failure was similar in groups 1 and 2 in our cohort, 5.2 vs 7.2 months respectively, however we used different cut-offs of 5-9 BMs and >10 BMs. The BMV at one year was similar in group 2 (11) to group 1 (9.7) and both would be classified as intermediate risk (35), however at last follow up, BMV was higher in the group with more than 10 initial BMs (2.7 vs 1.9). It is likely that the BMV and the irradiated volume are most predictive in combination. Technically, it is highly feasible to repeat courses of SRS, as we did for 57% of patients with subsequent new brain metastases, to effectively postpone WBRT (36). Generally, due to the spatial distribution of multiple small BMs, little consideration needs to be paid to the previous dose volume histograms (DVH), unless a metastasis is in close proximity to an organ at risk or to a previously irradiated metastasis due to the increased risk of radionecrosis associated with salvage re-irradiation (37). A contraindication to repeat SRS to new brain metastases would be leptomeningeal disease however, which may develop more frequently in patients with a higher number of brain metastases (38) (2/35, 5.7%, in our series) as these patients benefit from WBRT.

The acceptance of SRS as a safe technique for MBM prompted in silico comparisons of radiosurgery plans with multiple isocentres generated with conventional techniques (Gammaknife, Elekta) against linac-based volumetric arc radiation therapy (VMAT) as competing technologies (39). Due to the different beam geometry, greater low dose spill with VMAT was shown and the reporting of the gradient index (GI), in addition to the conformity index (CI), was recommended to compare dose to normal brain. The treatment planning software used in this study generates the GI as well as the inverse Paddick conformity index (PI) (40). **Table 2** shows that the CIs generated by the SI-DCA plans were similar to those achieved with a Gamma knife (Elekta, Sweden), as reported by Hazard et al. (15). In the case of MBM, if metastases are located close together, the dose GI (DGI) is inversely related to the distance between metastases and is affected by the accumulation of dose between metastases (41).

Although it is suggested that an ideal GI would be under 3 for a single lesion, as the PTV volume falls below a size of 0.5cm^3^, larger GI values must be expected. This is shown in a theoretical analysis of the dose spillage based on PTV surface area and volume and based on clinical data (42). **Table 2** shows GIs at the higher end of the range (3.8 in group 1 and 4.0 in group 2) which reflect that in the case of SRS for MBM, the PTV is typically very small and that the GI is increased due to close proximity of the metastases. However, importantly, it also shows that plan quality was not inferior for 10-15 brain metastases as compared with 5-9 brain metastases

**Figure 4:**
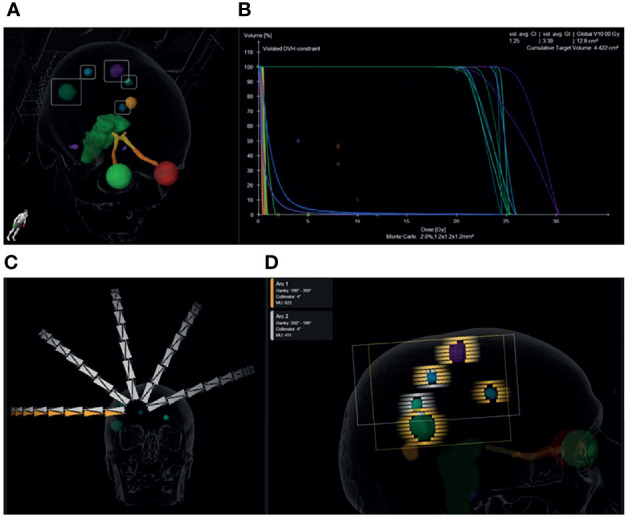
Graphical representation of a single isocentre dynamic conformal arc plan prescribed to 1 x 20 Gy for a patient with 7 brain metastases from non-small cell lung cancer. Five metastases were treated with this plan. The 6^th^ and 7^th^ were located inferiorly and were irradiated with 1 x 20 Gy in a second plan to maintain the distance of the PTVs from the isocentre below 5 cm. 3D view of the location and size of the metastases **(A)**. Dose volume histogram showing >99% coverage of the PTVs with 100% dose and minimal dose to the organs at risk. The cumulative PTV volume was 4.4cm^3^ and the cumulative volume of brain receiving 10 Gy is 12.8cm^3^. The mean conformity index for each PTV was 1.25 and the mean gradient index for each PTV was 3.39 **(B)**. 10 dynamic conformal arcs (5 duplicated non-coplanar arcs) were used to achieve the desired dose distribution **(C)**. 2.5mm MLC leaves were used to conform to the spherical metastases. Arc 1 (orange) treated 4 brain metastases along its path and Arc 2 (white) treated three on the return trajectory **(D)**.

Whereas SRS for MBM is technically feasible with multiple isocentres, the onerous treatment planning, quality assurance and the duration of therapy with irradiation of sequential targets impeded the wider adoption of the technique. SRS for MBM has been facilitated by the development of single isocentre (SI) techniques (43, 41), which are now available as time and resource-saving automated treatment planning software for the synchronous treatment of two or more BM (44). SI-VMAT plans have been compared head to head in dosimetric studies with plans generated with multi-isocentre VMAT (45, 46), multi-isocentre Gamma knife (39), multi-isocentre robotic radiosurgery (Cyberknife, Accuray) (47, 48), and with the SI-DCA technique used in our centre (**Figure 4**) and have been deemed clinically equivalent (49). SI-VMAT plans can be optimized to improve dosimetric parameters at the expense of number of arcs and MUs (50). A series of SI-VMAT plans for more than five brain metastases generated with 5mm MLCs reported comparatively poor GIs of 5.0 -5.6 (51). According to Ohira et al, optimization of the collimator angle (52) can achieve better sparing of healthy brain, although additional jaw tracking did not yield a benefit (53). The better GIs achieved with the DCA than with VMAT stem from its development as an extension of radiosurgery with conical collimators, but come at the cost of conformity in the case of non-spherical targets (54). We therefore prefer DCA for small brain metastases but instead use a more VMAT-like solution (Cranial SRS, Elements, Brainlab) for surgical cavities or elliptical lesions at the cost of a single isocentre. A clinician blinded to treatment technique did not find any significant difference in quality between plans generated with SI-VMAT (Hyperarc, Varian) and with RayStation for Cyberknife (16), reinforcing the notion that radiosurgery is platform independent as long as a high quality is achieved. Reviews of the technical aspects of SRS for MBM are available (55, 56) and guidelines for SRS for MBM with a linac (57) and with gamma knife (58) were published in 2019.

In addition to the choice between dynamic conformal arc or VMAT planning techniques, the width of the multileaf collimator (MLC) is a further variable to be considered. Use of a 2.5mm MLC as compared with a 5mm MLC for SI-VMAT results in significantly better CIs and GIs (52), although this can be somewhat offset by the addition of more VMAT arcs to 5mm MLC plans (59). This also applies to the DCA technique and is one of the reasons why we have grouped metastases and used more than one isocentre (**Figure 4D**) to ensure coverage by the high definition 2.5mm MLCs in the central 8cm of the field, rather than by the 5mm MLCs at the periphery. A major reason underlying the use of more than one isocentre in our patients to date has been concern about increasing rotational uncertainty with increasing distance from the isocentre and risk of compromise in coverage (7). Use of a head frame for SI-VMAT to reduce rotational uncertainties has been reported (60), however frameless linac-based SRS is more usual, and inaccuracies within 1mm for targets in phantoms within 6cm of the isocentre have been documented (61). An alternative approach is to increase the PTV margin with increasing distance from the isocentre to account for any uncertainties. However, a recently published series did not find that local failure correlated with increasing distance of the target from the isocentre using an image-guided frameless approach with patient positioning in 6DoF, a uniform 1mm PTV margin and a median distance to isocentre of 4.7cm (0.2-10) (62). A third reason for using more than one isocentre in our patients is that the SI technique could not combine different fractionation schedules. As mentioned above, a DCA plan is preferred for intact metastases and an hfSRT VMAT-like plan for surgical cavities or metastases near organs at risk such as the brainstem and chiasma. A further advantage of 2-3 SI plans is that different groups of brain metastases can be irradiated on different days, to spare normal brain through spatial fractionation (63). Whilst whole brain radiotherapy with hippocampal avoidance has been developed to reduce neurocognitive decline following the irradiation of MBM, the best way to minimise dose and thus protect the hippocampus (7) and all other functional areas of uninvolved normal brain, is through radiosurgery (64), even for more than 10 BMs (29). In one series, one third of patients had cognitive dysfunction before SRS (65) and such patients require efficient therapy without additional neurocognitive toxicity.

It is well established that the side effects of radiosurgery increase with the volume of a brain metastasis, hence the recommendation for resection or a dose reduction according to diameter (RTOG) or hypofractionation to minimise the risks of radionecrosis. In the setting of MBM, the irradiated volume will increase as the number of brain metastases increases. The metrics are being elucidated in parallel with the adoption of the technique, but current practice is to apply the 10cm^2^ V10Gy or 8cm^3^ V12Gy constraint to each metastasis as if treating a single metastasis (66). At present the dose is usually reduced according to the diameter of the largest metastasis (55) but is not known if the traditional RTOG constraints apply in the context of MBM and a review as to the possible approaches to dose prescription for adjacent metastases has recently been published (67). The low rates of radionecrosis here, according to contrast-enhanced MRI, are likely due to the small lesion size and use of hypofractionation in up to 30% of cases.

The Japanese JLGK0901 study showed no difference in cumulative complication incidence for patients with 5-10 BMs as compared with 2-4 BMs or a single BM (68) with a total cumulative volume of 15 cm^3^ (11, 68). It has become widely recognized that cumulative volume is more important than the number of metastases, however there is no current consensus as to maximum safe volume and a cut-off of 25cm^3^ is routinely used by another group (58). Volume not only plays a role in toxicity but also prognosis, as a cut-off of 7 cm^3^ irradiated volume was associated with a difference in overall survival of 20 vs 7 months in a series of patients with breast cancer (69). Tumor volume >10cm^3^ but not number of BMs has been associated with worse OS (12, 70) and a PTV <7.1 cm^3^ was the only significant prognostic factor for survival (64.1 vs 39.5% 1 year survival) in the series reported by Alongi et al. (30). When choosing a cumulative volume cut-off from the literature, it is important to consider the technique employed. For example, we have adopted an upper limit of 7 cm^3^ cumulative GTV as, with a 1mm margin to PTV, this equates to approximately 15 cm^3^, the cumulative irradiated volume recommended by Yamamoto et al. In our experience, this limit is more often reached in patients treated with SRS for fewer, larger symptomatic metastases than in patients with the numerous low volume metastases presented here. Of note, patients in group 2 had a median cumulative PTV of 7.2 cm^3^ and a maximum total PTV of 11.1 cm^3^.

In 2018, a survey of radiosurgery practitioners reported that 77% of respondents would offer SRS alone for 7 brain metastases under 1 cm diameter with extracranial disease control, 46% for 10 brain metastases, 26% for 15 brain metastases (71). The volume of brain metastases was deemed more important than the number and performance status was also a vital selection parameter. Nam et al. found recursive partitioning analysis (RPA) score (72) to be more important than multiplicity as did Salvetti et al, and all our patients had a Karnofsky Performance Index between 80 and 100% and an RPA of II. Regression analysis to compare groups 1 and 2 was not performed as the lack of events (0% local failure group 1, 0.02% local failure group 2, no reported toxicity) meant this analysis was unlikely to yield any data of significance. In 2021, a survey of the German Radiation Oncology Society, including but not exclusively radiosurgery practitioners, found that WBRT is still the most common modality used for 4-10 brain metastases, with SRS offered by a third according to performance status and number of metastases (73). These surveys highlight the current controversy regarding the optimal management of 5 or more BMs outside brain tumour centers with high volume of patients and the tendency of radiation oncologists to offer WBRT as compared with neurosurgeons practicing radiosurgery (58). Practice may change with the future publication of the current trials randomizing WBRT against SRS (74), however accrual is challenging as SRS is usually the patient’s preference and is often available off trial.

The main strength of this series is homogeneity: patient selection by one senior radiation oncologist led to very similar patient demographics in the two groups apart from the number of brain metastases. The delineation of organs at risk was standardized by automatic segmentation and target contouring by two experienced radiation oncologists minimized interobserver variability (data not shown). Predominantly automated treatment planning by two senior physicists contributed to the high quality plans. An internal guideline was followed to ensure consistent procedure, however the plan was individualized for each patient according to the distance of the brain metastases from the isocentre, the treatment prescription, fractionation and proximity to organs at risk. The main weakness is the limited number of patients, however most series originating outside Japan are of similar size. The median overall survival of 10 months for patients with 5-9 BMs is consistent with Yamamoto et al. (12) and Nichol et al. (75), and the median survival of in our small group of patients with 10 or more BMs exceeds that reported to date.

## Conclusion

Our data are consistent with the literature, which shows non-inferior intracranial outcomes for radiosurgery for 5 or more small volume brain metastases as compared with 1-4 brain metastases. Extracranial disease progression is the most common cause of death, even in patients with more than 10 brain metastases. Due to its high efficacy and low toxicity, SRS can be a cost-effective therapy for MBM and can be offered to patients with a good performance status and small volume intracranial disease with future therapeutic options for any extracranial disease. In view of the literature corroborating cumulative tumour volume being more prognostic than the number of metastases, prognostic scores should continue to be developed to optimize patient selection for this therapeutic modality.

## Data Availability Statement

The raw data supporting the conclusions of this article will be made available by the authors, without undue reservation.

## Ethics Statement

The studies involving human participants were reviewed and approved by Ethikkommission Nordwest- und Zentralschweiz. Written informed consent for participation was not required for this study in accordance with the national legislation and the institutional requirements.

## Author Contributions

All authors contributed to study conception and design. Material preparation, data collection and analysis were performed by SR, NL, SA, TL, and OR. The first draft of the manuscript was written by SR and all authors commented on previous versions of the manuscript. All authors read and approved the final manuscript.

## Conflict of Interest

SR has received Speaker’s Honoraria from Brainlab.

The remaining authors declare that the research was conducted in the absence of any commercial or financial relationships that could be construed as a potential conflict of interest.

## Publisher’s Note

All claims expressed in this article are solely those of the authors and do not necessarily represent those of their affiliated organizations, or those of the publisher, the editors and the reviewers. Any product that may be evaluated in this article, or claim that may be made by its manufacturer, is not guaranteed or endorsed by the publisher.
